# Protective Effects of Hydrogen against Low-Dose Long-Term Radiation-Induced Damage to the Behavioral Performances, Hematopoietic System, Genital System, and Splenic Lymphocytes in Mice

**DOI:** 10.1155/2016/1947819

**Published:** 2016-09-27

**Authors:** Jiaming Guo, Deyun Zhao, Xiao Lei, Hainan Zhao, Yanyong Yang, Pei Zhang, Pengfei Liu, Yang Xu, Meizhou Zhu, Hu Liu, Yuanyuan Chen, Yunhai Chuai, Bailong Li, Fu Gao, Jianming Cai

**Affiliations:** Department of Radiation Medicine, Faculty of Naval Medicine, Second Military Medical University, No. 800, Xiangyin Road, Shanghai 200433, China

## Abstract

Molecular hydrogen (H_2_) has been previously reported playing an important role in ameliorating damage caused by acute radiation. In this study, we investigated the effects of H_2_ on the alterations induced by low-dose long-term radiation (LDLTR). All the mice in hydrogen-treated or radiation-only groups received 0.1 Gy, 0.5 Gy, 1.0 Gy, and 2.0 Gy whole-body gamma radiation, respectively. After the last time of radiation exposure, all the mice were employed for the determination of the body mass (BM) observation, forced swim test (FST), the open field test (OFT), the chromosome aberration (CA), the peripheral blood cells parameters analysis, the sperm abnormality (SA), the lymphocyte transformation test (LTT), and the histopathological studies. And significant differences between the treatment group and the radiation-only groups were observed, showing that H_2_ could diminish the detriment induced by LDLTR and suggesting the protective efficacy of H_2_ in multiple systems in mice against LDLTR.

## 1. Introduction

As a basic part of treatment for many kinds of tumors, fractionated irradiation is extensively employed in the management of cancer victims [1]. In addition, with the nuclear technologies increasingly developed and utilized, the adverse effects of radiation exposure (especially the low-dose irradiation) on patients, occupational workers, astronauts, and even the public have been progressively noticed [2, 3]. As a result, it has drawn much attention of both researchers and the public to be more concerned with biological effects of the low-dose long-term radiation (LDLTR).

In fact, exploitation of the ideal radioprotectors has always been emphasized in the field of radiation while most of the radiation protectants currently being developed have certain shortcomings related to efficacy or toxicity [4, 5]. In 2007, Ohsawa et al. reported that H_2_ could selectively reduce cytotoxic reactive oxygen species (ROS) such as ∙OH and ONOO^−^ and exert therapeutic antioxidant activity in brain ischemic/reperfusion damage [6], after which a worldwide research climax on hydrogen was set off [5, 7–15]. In the field of radioprotection, our department demonstrated that H_2_ treatment protected both cultured cells and mice from high-dose acute radiation damage and exerted radioprotective effects on the gastrointestinal tract, cardiovascular system, immunocytes, and spermatogenic epithelium from gamma irradiation [12–15]. However, there is no report for the protection of LDLTR. This study is designed to investigate the effect of H_2_ on the adverse effects of LDLTR in mice.

As is known, ironizing radiation (IR) affects organism via producing free radicals and ROS, which then attack DNA, cellular membranes, and vital proteins resulting in cell death, apoptosis, and other types of functional damage in radiosensitive tissues such as bone marrow, reproductive organs, spleen, and peripheral blood cell parameters [16]. Moreover, though it is not quite sensitive to radiation, its damage on the central nervous system (CNS) deserves much emphasis as it could lead to behavioral disorders for certain groups of people such as patients with unavoidable head-direct radiation exposure therapy [1] and astronauts who have to perform some kind of special tasks [3].

In the present paper, we examined the body mass (BM) changes, behavior performances, chromosome aberration (CA), peripheral white blood cells count, sperm abnormality (SA), splenic lymphocyte transformation (LTT), and histopathological sections of radiosensitive tissues of BalB/c mice exposed to low doses of gamma radiation (0.1 Gy, 0.5 Gy, 1 Gy, and 2 Gy) for a long term (8 weeks) in a dose fractionation pattern (twice a week) and observed the definite competence of H_2_ to protect LDLTR-injured mice.

## 2. Materials and Methods

### 2.1. Animals

80 BALB/c adult wild-type male mice, weighing 20–25 g (7 Weeks old), were obtained from Shanghai Laboratory Animal Center of Chinese Academy of Science. They were maintained at 23 ± 1°C with free access to water and food, under a 12 : 12 h light/dark cycle (lights on at 07:00 hours). Mouse cages were replaced every two days and kept clean all the time. Before the experiment started, mice were allowed to accommodate to the new environment for a week. After that, the whole 80 mice were randomly divided into four radiation groups (0.1 Gy, 0.5 Gy, 1 Gy, and 2 Gy) and one sham radiation group (as control), each of which was additionally divided into two groups (hydrogen-treated group and nonhydrogen one, *n* = 8). The weight of each mouse was tracked every 3 days until the mice were sacrificed. All living conditions and tests were approved by the Second Military Medical University Institutional Animal Care and Use Committee and all the efforts were made to minimize animal sufferings.

### 2.2. Total-Body Irradiation

A ^60^Co irradiator was used for total-body ionizing irradiation. Considering the fact that the radiation durations were long and might place a more severe influence on the subjects and the radiation-delivered rates were so low that the effects of the variety were minor, unanaesthetized mice were placed in well-ventilated plastic boxes and exposed to the ^60^Co gamma radiation for 48 min per time synchronously twice a week for eight weeks. The distances of different radiation dose groups from the source were 6 m, 2.68 m, 1.89 m, and 1.34 m in accordance with total irradiation doses of 0.1 Gy, 0.5 Gy, 1.0 Gy, and 2.0 Gy, which were, respectively, delivered at dose rates of 0.13 mGy/min, 0.65 mGy/min, 1.3 mGy/min, and 2.6 mGy/min. Every time after the radiation exposures, mice were immediately removed from the plastic box and allowed free access to food and water. Finally, 1 week after the last time of radiation, the behavioral tests were performed and the other test end points followed subsequently.

### 2.3. Hydrogen-Rich Water Preparation and Administration

After the accommodating period, the experimental mice were allowed free access to normal double distilled water (the vehicle group) or hydrogen-rich water (hydrogen-treated group) which was made by adding a hydrogen-rich water generator (Shanghai Shes Green Technology Co. Ltd.) into double distilled water to persistently produce hydrogen, in which way the mice were housed until sacrificed. To keep the H_2_ concentration at an acceptable level (at least 0.1 mM), the generator was cleaned at least once a week according to the instruction manual of the product and the H_2_ concentration was confirmed using a needle-type H_2_ sensor (Unisense, Aarhus, Denmark).

### 2.4. Behavioral Tests

One week after the last time of radiation exposure, the behavioral tests, consisting of both forced swimming test (FST) and open field test (OFT), were carried out immediately. All tests were conducted on separate days with each test period being one day apart and strictly conducted during the dark cycle.

The FST was carried out based on the method previously described by Dou et al. with minor modifications [17]. Briefly, mice were dropped one by one into cylinders (height 40 cm, diameter 30 cm) which were filled with water to a depth of 30 cm for 6 min, maintained at 25 ± 1°C. In such a situation, from which they cannot escape, the mice quickly turned to be immobile, floating in a vertical position, and making only small movements to keep their heads above water. Behaviors were monitored from above by a video camera for the following analysis. The total duration of immobility/mobility was scored.

We evaluated the anxious behavior of the subjects in an open field test (OFT) as described elsewhere [18, 19]. It is based on a behavior analysis of rodents exposed to an unfamiliar arena during a determined period. In detail, OFT activity was assessed during 5 min using the EthoVision XT 8.5.610 automatic system (Noldus Information Technology, Wageningen, Netherlands). The apparatus consisted of a plastic box measuring 60 cm × 60 cm × 50 cm, of which the central 30 cm × 30 cm area was defined as the open field (OF) center zone, with a peripheral area 15 cm in width considered as outer area of the OF. The following measures were taken: total moved distance, mean velocity, total time spent in the central zone, and entries into the central zone. The testing room was dimly lit by a red lamp with luminosity between 10 and 25 lux.

### 2.5. Chromosome Aberration

The bone marrow chromosome aberration (CA) investigation was conducted as reported previously with minor alterations [20].

*Bone Marrow Collection*. Colchicine (at a final concentration of 400 *μ*g/mL) was administered by intraperitoneal (i.p.) injection into each mouse (3 *μ*g/g body weight) 3.5 h prior to bone marrow collection. Bone marrow was flushed from every left femur with phosphate buffered saline (PBS). Then, the PBS bone marrow mixture was centrifuged at 1800 rpm for 8 min at 4°C, and the cell concentrate was placed into an centrifuge tube containing 7 mL 0.075 M KCl, mixed and incubated for 25 min at 37°C. After incubation, 1 mL fresh Carnoy's solution (3 : 1 methanol : acetic acid) was added to the tube bottom, carefully mixed and centrifuged at 1000 rpm for 10 min. Then the supernate was discarded and the cells were incubated with another 5 mL fresh fixative for 20 min and centrifuged as mentioned above for two more times. Finally the supernatant was decanted, with a little of it remaining (about 0.15 mL). Then the cells were mixed and dropped onto clean, wet cold microscope slides which had been placed at 30° angle of inclination to horizontal plane with a height of 80 centimeter. Slides were allowed to dry with the swift heat of an alcohol lamp. 

*The Staining of the Slides*. Because of the simple and rapid procedure, the conventional Giemsa staining method was suitable for the analysis of large numbers of chromosome metaphases of the irradiated mice. Slides with bone marrow metaphase cells were stained with 10% Giemsa for 15 min, rinsed, air-dried, and mounted with a glass cover slip. 

*Scoring of Chromosome Aberrations*. The slides were coded and blinded and observed under a light microscope (Olympus Bx 51, Olympus, Tokyo, Japan) connected to a chromosome aberration analysis system (CytoVision 3.93, Applied Imaging Corp., 120 Baytech Drive, San Jose, CA 95134-2302, USA) that was employed for this experiment. At least 100 metaphases per each mouse were scored and 8 mice were included in each group. Chromosome aberrations observed in bone marrow metaphases were scored as follows: (1) chromosome- and chromatid-type fragments, identified as acentric fragments that derived from a chromosome or chromatid severance; (2) chromatid (or isochromatid) gaps, identified as an a chromatic break along the length of a chromatid of which the length was less than the width of the chromatid; (3) dicentrics, identified as a chromosome (or chromatid) end joined to another chromosome (or chromatid); and (4) Robertsonian translocations, identified as two chromosomes joined at their centromeres; (5) deletion, defined as a chromosome with centromere lacking a chromatid piece so it has less than the size of a normal mouse chromosome and dissymmetry; (6) exchanges, identified as the intertranslocation of two chromatid fragments. All metaphases were scored in the same fashion to enable consistent comparisons.  Figure 5 shows representative photomicrographs of metaphase bone marrow cells from each treatment group. After scoring, the slides were unblinded and results were compiled according to the respective treatment group.

### 2.6. Peripheral Blood Cells Parameters Analysis

For the study of hematological parameters, blood samples were drawn from angular vein venous into the marked anticoagulant tubes just before the animal was sacrificed (10 days after the last time of radiation) and were sent to be analyzed by an automatic blood cell analyzer (Siemens, ADVIA 2120i, Siemens Healthcare Diagnostics Inc., Tarrytown, NY 10591-5097 USA) in the Laboratory Animal Center of our college as soon as possible. A five-classification result was obtained and all of the indexes were analyzed [21].

### 2.7. Sperm Abnormality

Reproductive toxicity was tested by applying sperm-shape abnormalities assessment using the methods described elsewhere with some modifications [22, 23]. 

*Sperm Collection and Handling*. Murine sperm were collected from the epididymides of both sides as described elsewhere. Briefly, epididymis (free of fats, vas deferens, and other tissues) from both sides of testis of mice was dissected out, cut into 3-4 parts by a pair of clean scissors, and immediately placed into 1 mL PBS at 37°C. After standing for 20 minutes when most of the inner content (spermatozoa mainly) had swum out into the saline, the mixture was then thoroughly shaken, was filtered through a 200-mesh sieve, and made the target sperm suspension. All steps above were made at 37°C, in order to minimize the damage to the sperms. 

*The Preparation and Staining of Slides*. The homogenate drops on the slide were smeared smoothly by one side of another clean slide, allowed to air-dry, fixed by methanol for 5 min, and stained by Eosin solution (1%) for one hour. Finally, these slides were rinsed slightly and air-dried and examined by microscopy for the presence of various sperm-shape abnormalities. 

*Sperm Morphology Observation*. The technique of Wyrobek et al. [24] was adopted for sperm abnormality study with minor modifications. Sperms were analyzed at 400-fold magnification using a light microscope (Olympus Bx 51, Olympus, Tokyo, Japan) and scored as (A) normal sperm; (B) sperm with banana-shape head; (C) sperm without-hook head sperm; (D) gigantic head sperm with an evidently larger and fatter head; (E) sperm with double heads; (F) sperm with two or more tails; (G) sperm with a bent tail; and (H) sperms with an amorphous head. A minimum of 1000 sperms were counted per mouse. In addition, the mean differences of abnormal spermatozoa populations between groups were compared using a two-way ANOVA method (*p* ≤ 0.05 was considered significant).

### 2.8. Lymphocyte Transformation Test

The lymphocyte transformation test (LTT) was performed in order to determine whether there were distinctions in the splenic T cells' proliferation viability between H_2_ treated and no-H_2_ treated mice. 

*Preparation of Lymphocyte*. The lymphocyte transformation test was conducted as described previously with minor modification [25]. Briefly, spleens of BALB/c mice were removed aseptically and placed into 3 mL Hank's solution (Biotop) on a small sterile dish, ground, and filtered through a 200-mesh sieve. Then, the suspension was centrifuged (1200 rpm, 7 min) and the supernate was discarded. Lysate of Tris-NH_4_Cl kept at 37°C was added into the mixture and allowed to stay for 5 min to lyse the red blood cells (RBC) thoroughly. Next, the relatively purified lymphocytes were obtained and washed with Hank's solution and finally suspended in RPMI 1640 medium containing 10% fetal bovine serum (FBS) and 1% antibiotic (100 U/mL penicillin and 100 mg/mL streptomycin). 

*Assessment of Cytotoxicity by cck-8 Assay*. To assess the viability of spleen T lymphocytes, we performed cck-8 assay, a test of metabolic competence predicated upon the assessment of mitochondrial performance [26]. For these assays, 1 × 10^6^ BALB/c mouse spleen cells in 0.1 mL were incubated with (*S*: stimulated wells)/without (*NS* wells: no-stimulation control wells) concanavalin A (Con A, TypeIV, Biosharp) in a concentration of 20 *μ*g/mL for 48 hours, to get the T lymphocytes stimulated. That was followed by the harvest of the cells, with cck-8 stock solution (10 *μ*L each well) applied to each of the wells for 4 h, while the control wells (*C*: wells without cells) were treated with only RPMI 1640 medium. At last, the absorbance of formazan crystals dissolved in each well was measured at 450 nm using a Microplate Reader (TECAN, infinite F50, Tecan Group Ltd., Seestrasse 103,8708 Männedorf). The optical density (OD) of the formazan generated in control cells that were treated with RPMI 1640-only medium was considered to represent 100% viability. The capability of proliferation of mice splenic cells was calculated by the formula below:

(1) $$\begin{array}{c} \text{Proliferation capability}\left(\;\%\;\right)=\frac{\left[S-C\right]}{\left[NS-C\right]}\times 100, \end{array}$$

where *S* is the OD value of wells with cells, Con A and cck-8 solution. *C* is the OD value wells with cck-8 solution, RMPI 1640 medium, but without cells or Con A. *NS* is the OD value wells with cells, cck-8 solution, but without Con A.

### 2.9. Histopathological Studies

Testis and femoral bones (one for each mouse) were removed and fixed with 4% paraformaldehyde. Then the tissue samples were embedded in paraffin and cut into serial sections of 4 *μ*m thick. The histopathological discriminations were performed on tissue sections after hematoxylin-eosin (H&E) staining as it was described previously [16, 27, 28].

### 2.10. Statistical Analysis

This experiment was contrived based on the factorial design. All data were expressed as mean ± standard deviation (SD) of the 8 animals in each group. For the determination of significant intergroup differences, two-way analysis of variance (ANOVA) across radiation dose and treatment (hydrogen or not) was used followed by Fischer's LSD post hoc comparisons where appropriate to show differences between groups, unless specially stated otherwise. A* p* value of less than 0.05 was considered statistically significant. In addition, Levene's test for equal variances was utilized to test for homogeneity. SPSS Software, version 21 (SPSS Inc., Chicago, IL, USA) was utilized to all statistical analyses. OriginLab Software, version 9.0 (OriginLab Software Inc., Massachusetts, USA) was used to make the figures.

## 3. Results

### 3.1. H_2_ Restricted the Abnormal Trend of Body Weight Overincrease Caused by LDLTR

**Figure 1:**
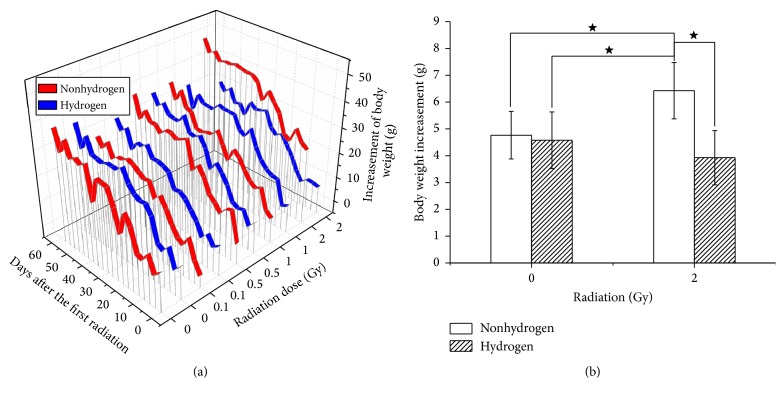
3D Ribbons chart giving an overview of body mass change of all groups (a). As we can see from Figure 1, all of the groups gained steadily increasing body weight over the days. No notable difference was found in the 0.1 Gy and 0.5 Gy group. But as for both the 1 Gy and 2 Gy groups, we found that H_2_ could restrict the excessive growth of the body weight. (b) Showed the comparison of accumulation of the body weight gain between the sham and 2 Gy radiated groups either treated with H_2_ or not (^★^ *p* < 0.05, two-sample *t*-test).

The 3-dimension Ribbons figure (Figure 1(a)) shows an overview of the trend of body weight gain due to the LDLTR and H_2_ treatment. Body weight increased smoothly (especially for the H_2_ groups) as days elapsed in a normal range all over the groups except the higher dose groups (1 Gy and 2 Gy) without H_2_ treatment. In addition, H_2_ showed a slight ability to decrease the weight gain all through the lower-dose IR groups (0.1 Gy and 0.5 Gy). Moreover, we found that after a period of radiation exposure (3 weeks) the non-H_2_ treated mice in both the 1 Gy (dose rate: 1.3 mGy/min) and 2 Gy (dose rate: 2.6 mGy/min) IR groups were gaining greatly more body weight than the H_2_ treated ones. And the comparison of this kind in the 2 Gy dose group is shown in Figure 1(b).

### 3.2. H_2_ Improved the Anxious and Depressive Performances Caused by LDLTR

#### 3.2.1. Open Field Test

**Figure 2:**
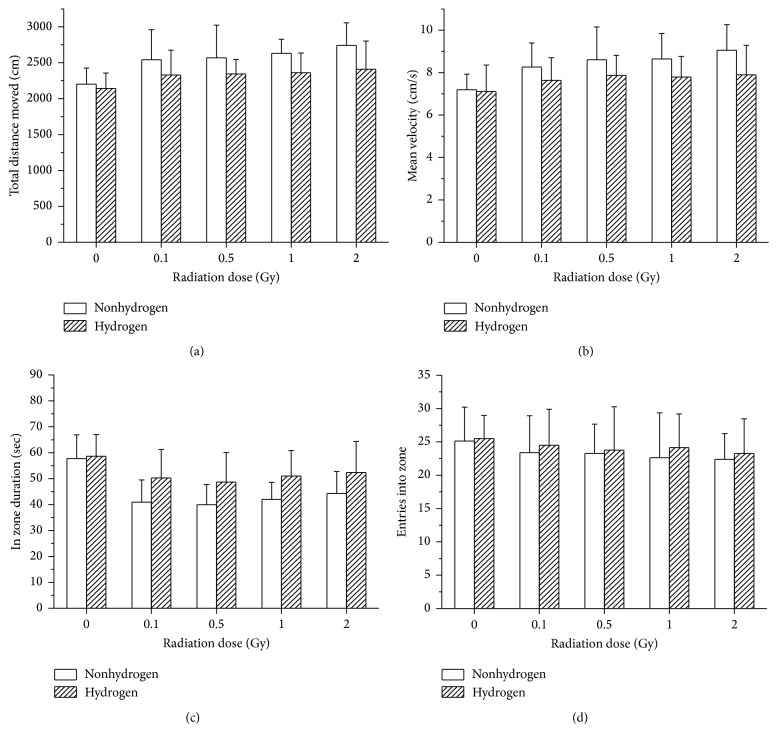
BalB/c mice, either orally ingested with hydrogen-rich water or not, were tested in an open field arena after 8-week whole-body irradiation, for which two-way ANOVA was utilized. Mice treated chronically with hydrogen-rich water displayed a reduction in anxiety-like behaviors, travelling less distance in total ((a), *F* = 9.617, *p* = 0.003), moving at minor velocity ((b), *F* = 7.096, *p* = 0.01), and spending more time within the central zone ((c), *F* = 5.552, *p* = 0.021) compared to mice drinking normal water. They also performed slightly higher (not statistically) entries into the zone (d). However, except for the comparison of anxiety-like behaviors between 0 Gy group and any other dose group, no significant difference was detected between various radiation doses. And as for all the behaviors observed above, there was no evident interaction between the effects of radiation dose and hydrogen as well. Data represent mean ± SD. Significant difference (*p* < 0.05) based on two-way ANOVA with post hoc comparison (LSD-*t*). *N* = 8.

In the open field test, as for the total distance moved, the velocity of moving, and the central zone entry frequency, our data indicated that subjects with all doses performed significantly differently from the sham irradiated ones, while no notable difference was found between varying-dose-radiated groups (Figures 2(a), 2(b), and 2(c)). In detail, mice treated with hydrogen-rich water moved a smaller distance than the vehicle group (Figure 2(a)). Moreover, a significant decrease in velocity was observed in the H_2_ treated groups than the non-H_2_ ones (Figure 2(b)). Additionally, hydrogen-treated mice spent more time in the center of the chamber (Figure 2(c)). However, a merely slight (not significant) descendent trend of zone entries was found as the radiation dose rose, and H_2_-rich water did not seem to quite increase the entries frequency either (Figure 2(d)).

#### 3.2.2. Forced Swimming Test

**Figure 3:**
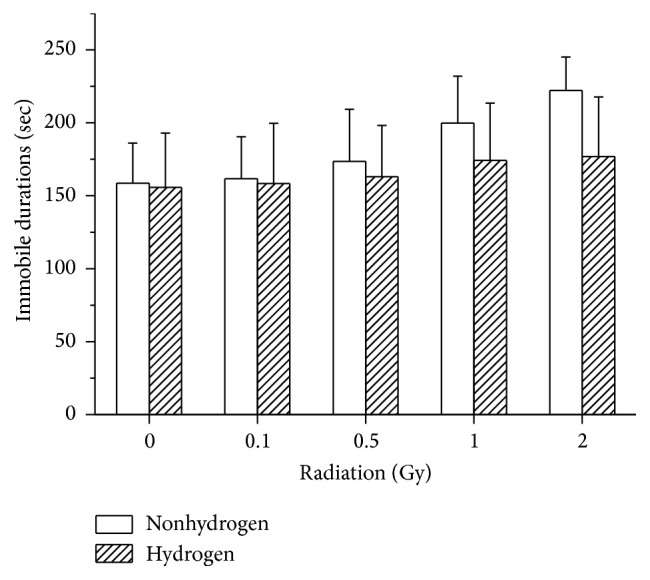
Effects of oral administration of H_2_-rich water and series of doses of radiation on the immobile durations in the FST in mice. H_2_ treatment decreased the immobility time significantly according to two-way ANOVA (*F* = 4.449, *p* = 0.038). Values were expressed as mean ± SD (*n* = 8).

Our results demonstrated that H_2_ treatment reduced the immobility time in the FST (Figure 3, *F* = 4.449, *p* = 0.038). The immobility time increased after irradiation in a dose dependent manner (*F* = 3.873, *p* = 0.007). However, there was no significant interaction between H_2_ treatment and radiation exposure.

### 3.3. H_2_ Benefitted the LDLTR-Damaged Hematopoietic Tissues and Cells

**Figure 4:**
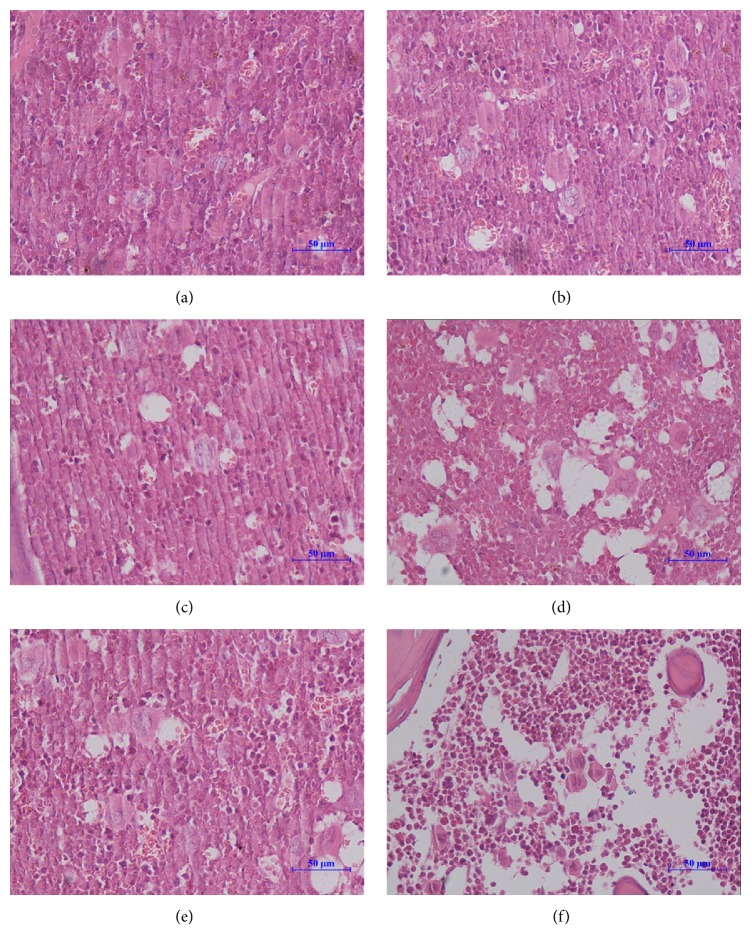
H&E stained representative bone marrow sections. Nonradiated group whether handled by hydrogen (b) or not (a) showed active hematopoiesis. As the radiation doses ascended ((d) 1 Gy, (f) 2 Gy), the hematogenesis was attenuated represented by the decreased cellularity of nucleated cells and the increased number of vacuoles. However, in cases of hydrogen ((c) 1 Gy, hydrogen; (e) 2 Gy, hydrogen), hematopoietic cell density became almost as high as the vehicle group. Pictures were taken randomly at 400-fold magnification. Scale bar = 50 *μ*m.

**Figure 5:**
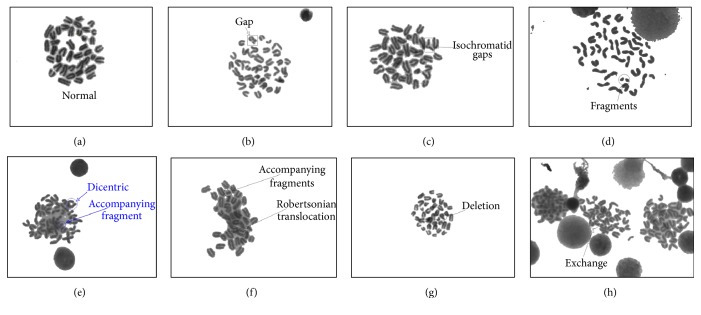
Photomicrographs of metaphase bone marrow cells 1 week after irradiation. Different types of aberrant chromosomes are shown here. (a) Normal metaphase, (b) chromatid-type gap, (c) isochromatid gaps, (d) chromosome fragments, (e) chromosome with dicentrics and the accompanying fragments, (f) Robertsonian translocation, (g) chromosome deletion, and (h) chromosomes with exchanged fragments. Arrows indicate specific aberrations. Pictures were taken at 1000-fold magnification with an immersion objective.

Pathological examinations of bone marrow after total-body irradiation (at the 1 Gy and 2 Gy dose level) showed that hydrogen administration effectively ameliorated the radiation-induced reduction of bone marrow cells (Figure 4), while at the 0.1 Gy or the 0.5 Gy dose no distinguishable changes were detected. This effect was consistent with a decrease trend in the chromosome aberration enhanced by irradiation in bone marrow cells. Moreover, the beneficial effects of H_2_ on reducing chromosomal radiation injuries appeared at dose level as low as 0.1 Gy (Figure 5, Table 1).

### 3.4. H_2_ Improved the LDLTR-Induced Injuries of the Peripheral White Blood Cell and Lymphocyte Count

**Figure 6:**
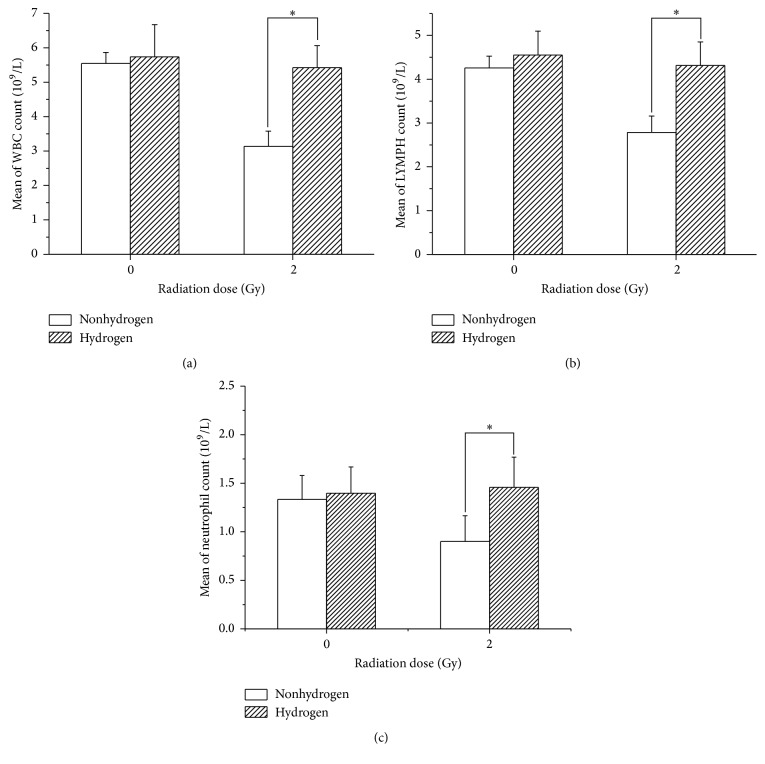
Results of peripheral WBC count, lymphocyte count, and neutrophil count of sham and 2 Gy irradiated mice, H_2_ treated or not. (a) Comparison of WBC count. (b) Display of lymphocyte count in different groups. Compared to the 2 Gy gamma radiation-induced cells counts, the H_2_ group had conspicuous enhancement effects both for the WBC (*t* = −2.948, *p* = 0.012,) and for the lymphocyte (*t* = −2.342, *p* = 0.037). An independent samples *t*-test (two tails) was used here. Asterisk notes that there existed a significant difference (*p* < 0.05) between the curve-connected groups. Values were expressed as mean ± SD (*n* = 8).

Compared to the sham-radiated ones, the mice from 2 Gy irradiated group were found to contain obviously less WBC and lymphocytes in their peripheral blood, which was greatly improved by H_2_ treatment (Figures 6(a) and 6(b)). However, in the terms of 1 Gy and less doses, the cell counts of both kinds seemed to be even more than the sham-radiated mice, and no protective effects of H_2_ were found.

### 3.5. H_2_ Improved the LDLTR-Induced Injuries of the Sperm Morphology

**Figure 7:**
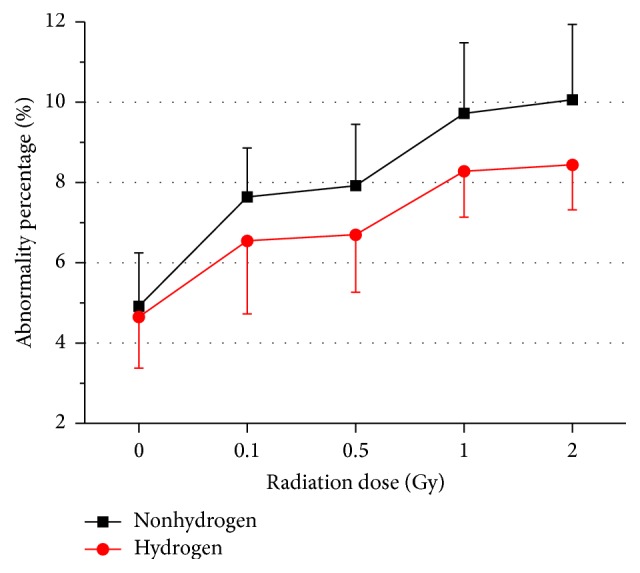
Effects of long-term oral application of hydrogen-rich water on the abnormalities percentage of sperm of subchronic different doses-radiated mice. Values were expressed as mean ± SD (*n* = 8).

**Figure 8:**
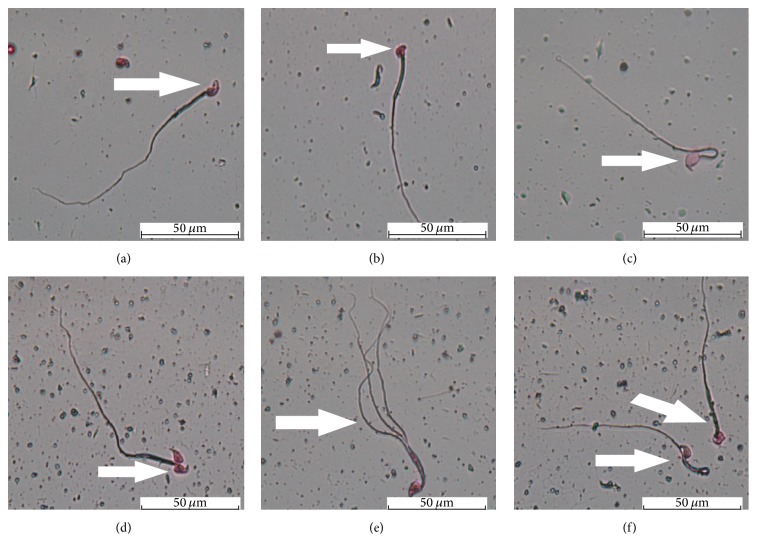
Types of sperm-shape abnormalities found in normal and radiated mice. (a) Sperm with banana-shape head; (b) without-hook head sperm; (c) gigantic head sperm; (d) sperm with double heads; (e) sperm with 3 tails; (f) sperms with a bent tail (the left) and amorphous head (the right). Scale bar = 50 *μ*m. (Magnification ×1000.)

Reproductive toxicity of LDLTR and its radioprotection of H_2_ were evaluated by employing sperm abnormality assays [22]. Morphological examination of bilateral epididymal sperm showed that sperms with abnormalities increased as the IR dose increased (Figure 7, *F* = 9.551, *p* = 0.000). Meanwhile, the elevated percentage of sperm abnormalities was decreased at every radiation dose group in the case of hydrogen (Figure 7, *F* = 4.74, *p* = 0.036). Nevertheless, no interaction across H_2_ treatment and radiation exposure was identified (*F* = 0.201, *p* = 0.936).  Figure 8 showed the different types of observed abnormalities, which included sperms with amorphous head, with double (multiple) head, without-hook head, with banana-shape head, with gigantic head, with double (multiple) tails, and with coiled tails.

### 3.6. H_2_ Improved the Testicular Microstructure Injured by the LDLTR

**Figure 9:**
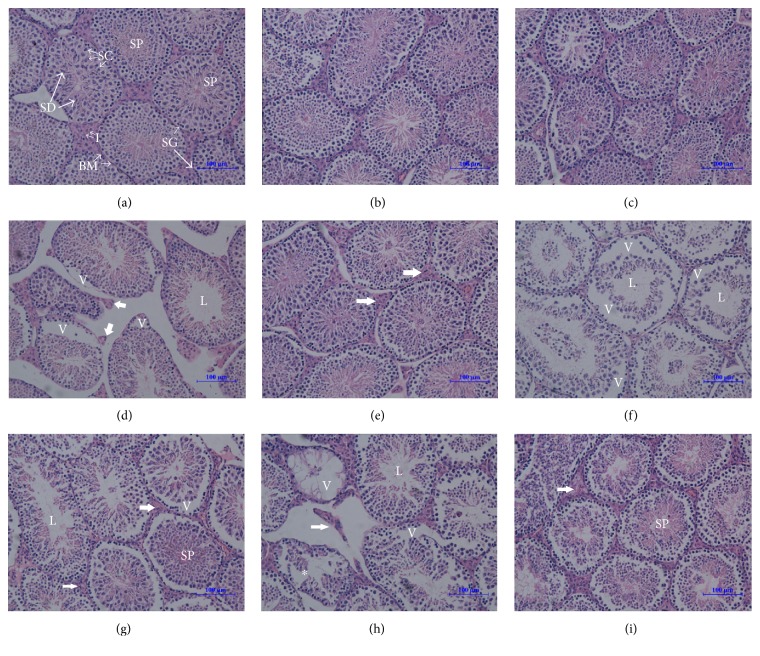
Photomicrograph of mouse testes after different doses of radiation and hydrogen treatment. Sections of adult mouse testes were stained with hematoxylin-eosin (H&E) staining. For the vehicle mice (0 Gy, nonhydrogen, (a)), the testes showed normal testes histoarchitecture with successive stages of spermatogenesis which include spermatogonia (SG), spermatocytes (SC), different stages of spermatids (SD), and spermatozoa (SP) surrounding a central lumen, and interstitial cell (I) and basal membrane kept their normal morphology as well. In addition, no apparent damage was detected in the 0.1 Gy dose groups, whether treated with hydrogen (c) or not (b). As low as 0.5 Gy radiation (d) was able to cause a disrupted organization (V: vacuole), the absence of spermatozoa (L: lumen), reduction of the depth of spermatogenic epithelium, and decreased interstitial tissue (thick arrow). However, tubular structure was restored to be nearly normal in the case of 0.5 dose-radiated with hydrogen-treatment groups (e), in which the interstitial cells increased apparently (thick arrow). For male BalB/c mice seminiferous tubule, more severe damage occurred with 1 Gy dose radiation (f), with a much decreased cellular density, and disorganization and vacuoles almost all over the tubules. By contrast, in cases of hydrogen (g), cellular density was elevated, as well as interstitial cells and spermatozoa. Testicular tissue of mice receiving 2 Gy doses of irradiation showed severe degenerative changes involving the majority of the seminiferous tubules characterized by shrunken, disorganized seminiferous tubules (indicated with an asterisk), severe and large vacuoles, and scarce interstitial cells. The seminiferous tubules were almost devoid of spermatozoa. Amazingly, testis from hydrogen-treated 2 Gy irradiated mice showed milder histological alterations with almost normal spermatogenic cycles. Hydrogen eliminated most of the morphologic alterations above, raising the cell density, sperms, and even the interstitial cells as much, though the diameters of the tubules were reduced and the vacuoles lessened slightly as well. (H&E: ×200. Scale bar = 100 *μ*m.)

Light microscope examination of sections of the testes from the control mice displayed testicular tissue with normal histoarchitecture that consisted of well-organized seminiferous tubules with intact spermatogenesis including spermatogonia, spermatocytes, different stages of spermatids, and spermatozoa surrounding a central lumen. Testicular cells density was high and interstitium was affluent (Figure 9(a)). Apart from 0.1 Gy radiated group, testicular sections from 0.5 Gy and 1 Gy to 2 Gy irradiated mice showed pathological alterations in tubular architecture when compared to those of the control mice. 0.5 Gy irradiation caused damage in the seminiferous tubules represented by the reduction of interstitial cells, presence of vacuoles, and absence of sperms in some tubules (Figure 9(d)). In comparison, mice receiving the same dose of irradiation and drinking hydrogen-rich water at the same time displayed a nearly normal tubular architecture with notably increased interstitium (Figure 9(e)). Testicular tissues of mice that received radiation of 1.0 Gy showed severe generative changes of the majority of the seminiferous tubules, with large vacuoles and vacant lumens (Figure 9(f)). However, in cases of hydrogen, both vacuoles and vacant lumens became less and smaller (Figure 9(g)). Furthermore, most of the seminiferous tubules were severely damaged and apparent vacuoles occurred accompanied by seminiferous tubules of smaller sizes in mice radiated with 2 Gy dose. In addition to vacuoles and lumens, sever disorganization both in and between tubules was found (Figure 9(h)). Though H_2_ treatment could not reverse the radiation-induced degenerative changes, it did reduce the damage to microarchitecture and maintenance of reasonable numbers of germ cells (Figure 9(i)).

### 3.7. H_2_ Elevated the Lymphocyte Activation Rate Damaged by the LDLTR

**Figure 10:**
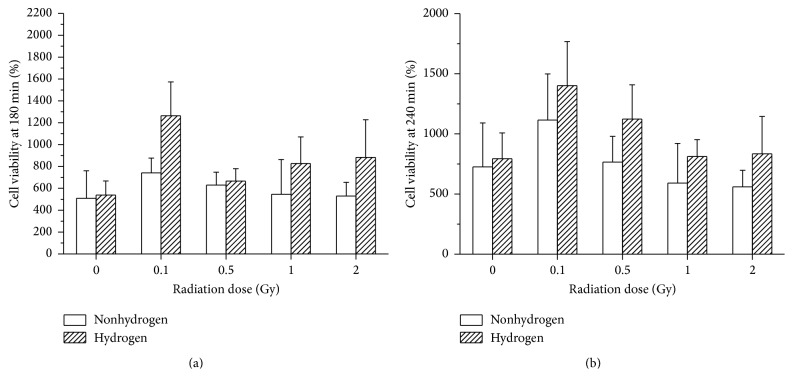
The cell viability of mice spleen was determined 180 min (a) and 240 min (b) after the addition of cck-8 solution. 180 min: radiation, *F* = 2.613 and *p* = 0.046; hydrogen, *F* = 5.075 and *p* = 0.029; 240 min: radiation, *F* = 3.438 and *p* = 0.015; hydrogen, *F* = 4.415 and *p* = 0.041.

The LTT showed that, whether at the 180 min time point or at the 240 min, the very low dose of radiation (0.1 Gy and 0.5 Gy) elevated the cell viability (Figures 10(a) and 10(b)); by contrast, when the dose was raised up to 2 Gy, the competence of transformation of the cells decreased (Figure 10(b)). However, no matter how the variety of the radiation doses influenced the cell viability (improved or damaged), the presence of H_2_ unanimously ameliorated the splenic cell in all the groups (Figures 10(a) and 10(b)).

## 4. Discussion

Here, we studied the bioeffects of H_2_ on LDLTR-exposed mice. We utilized a fractional radiation pattern for 8 weeks to simulate the subchronic low-dose IR exposure which is more common than the serious nuclear accident. Many kinds of methods were adopted in our experiment involving nutriology, hematology, genesiology, immunology, and even ethology aiming at detecting the comprehensive bioeffects of H_2_ and the LTLDR. In addition, we chose the two-way ANOVA to explore whether there was any interaction between H_2_ and some of the radiation performed (the four sorts of LDLTR).

Body weight is an indicator that can reflect the general condition of individuals. Meanwhile, overweight is regarded as a signal of being under stress [29]. Thus, we investigated the body weight gain consecutively throughout the experiment. Our results indicated that when exposed to 1 Gy or 2 Gy (both can exert harmful effects to organism) the subjects turned to put on weight too much, while H_2_ could lessen them to be normal. This was consistent with our OFT results which showed an antianxiety effect of H_2_ as well. As a well-known stressor, radiation of higher level (1 Gy or 2 Gy dose group in this study) can activate a series of in vivo responses including hormone alterations [29, 30] and ROS increase. H_2_ may normalize the body weight by adjusting the unbalanced hormone distribution or selectively scavenging the harmful ROS [5], but the concrete mechanism remains to be explored. Some previous studies reported that H_2_ could improve lipoids metabolism disturbance [23, 31], which may present a potential study direction for this mechanism.

As the OFT (open field test) is widely used to measure the anxiety level and assess unconditioned avoidance of fearful situations [32, 33], it was adopted by us for the assessment of mice anxiety as well. Moreover, the FST, as one of the most reliable and available models, is also regarded as one of the most frequently used tools for assessing depression level of the selected animal models, which has been reported to be believable among different laboratories [33]. In previous studies, larger doses of gamma rays, no matter cranial radiation or whole-body radiation, could alter the emotional distress state evidently [1, 32, 34], which was called as relieving methods. In our study, we found in the OFT that the low-dose radiation could also elevate the anxiety level by increasing the moved distance and the velocity of the mice and minifying the central duration of them. Simultaneously, H_2_ treatment was able to reduce the fearful level. Additionally, in the FST our study demonstrated that H_2_ could reduce the radiation-elevated immobile time fairly, which indicates an antidepressants-like effect of H_2_ on radiation-induced depression. To sum up, the LDLTR in our study affected the behavioral performances of the mice, including higher anxiety level and depression level, which could be mitigated significantly by H_2_ treatment. As to the possible mechanisms by which H_2_ provides the above beneficial effects, Liu et al. found that H_2_ intake was able to attenuate brain injury in mice, of which the process may be associated with the decreases of apoptosis, oxidative stress, and inflammation as well as upregulation of Nrf2/ARE signal pathway [35]. However, the exact mechanisms still need to be deeply explored in the future.

The hematological system is deemed as the most sensitive target organs of irradiation, of which the decrease of peripheral leukocytes is considered as the most classic indicator. As we know patients who are damaged by ironizing radiation will go through a severe period of peripheral blood parameters fluctuation and the most urgent treatment they need is to improve and restore their blood dysfunction. Moreover, the exposure of whole-body low-dose irradiation causes damage in bone marrow cells [36–38]. In addition, spleen is not only one of the most important hematopoietic organs, but also an immunity peripheral organ, for which the observation of splenocytes' performance is another important aspect as a marker for radioprotective efficacy [39, 40].

At first, WBC, lymphocyte, and neutrophil in the 2 Gy radiation group were found decreased compared with the other dose groups, and the inhibition was ameliorated by H_2_ greatly (Figures 6(a), 6(b), and 6(c)), showing the beneficial effects of H_2_ on the peripheral blood radiation damage. However, there still seemed to be some “contradicting findings” here. Mice radiated by 0.1 Gy, 0.5 Gy, and 1 Gy gained even higher WBC, lymphocyte, and neutrophil counts, which was apparently not affected by H_2_ treatment at all. But, in our opinion, this phenomenon can be explained exactly by the famous two special effects (adaptive effects and stimulating effects) which are excited on the condition of the “very low dose radiation” [41–43]. Therefore, it was concluded that our results reflected a comprehensive influence of all the various bioeffects of LDLTR. Nevertheless, the shortage of this study is that it was unable to distinguish the different bioeffects of the LDLTR separately and quite exactly. What is more, the mechanism of this process still remained to be determined.

Evaluation of the bone marrow CA is considered as a classic method of assessing hematopoietic genotoxic effects for it is directly relevant to real mutations [44]. The exact mechanism of the complicated process of CA forming is not entirely clarified yet. Nonetheless, we summarized several common points leading to structural CA according to the prevailing theories here: repressed synthesis of DNA, replication with an injured DNA template, and direct DNA breakage [44]. Our data indicated that H_2_ exerted radioprotection against LDLTR-induced CA, while H_2_ itself did not work on the bone marrow cells. The approximate linear quadratic dose response (the linear chart was not shown) for CA induced by irradiation was in keeping with former reports [45, 46]. One possible explanation for this radioprotective effect of H_2_ can be attributed to its ability to selectively scavenge free radicals that contribute to the production of CAs [6]. What is more, it was also reported that H_2_ could regulate some genes involving signaling pathways in cells, but the underlying mechanism still remains unknown. As for the morphological examinations, an all-inclusive indicator that gave us an overview of the bone marrow hematogenesis, the beneficial influence of H_2_ was detected merely in the 1 Gy and 2 Gy dose group. This is also consistent with the findings in the CA experiment. That is, in the cases of 0.5 Gy or lower, radiation-induced damage cannot be seen in the pathological slides. On the other hand, pathological slides views reflect a final complex action of overall relating bioeffects of the LDLTR including both damaging effects and stimulating activation for the marrow cells, which is susceptible to compounding factors, while the CA assessment was aimed at and focused on the aberrations of chromosome resulting from DNA impairments, so it is reasonable for the appearance of the contradiction for the two indicators in the 0.5 Gy or lower doses groups.

With quite hypersensitivity to radiation, spleen is held as one of the most important hematopoietic organs [39]. From the LTT results, two notable points can be drown here: (a) compared to the vehicle group, exposure to the very low dose (0.1 Gy and 0.5 Gy) of radiation enhanced the splenocytes viability (Figures 10(a) and 10(b)), while 2 Gy irradiation represses the transformation rate of the cells (Figure 10(b)); (b) aside from the sham-radiated groups, throughout all the dose level, H_2_ showed the competence of improving effects on the transformation rate. As mentioned above, the very low-dose radiation can place an influence of stimulation to the organism, especially the immunocytes such as lymphocytes in the spleen. Thus, our findings also confirmed this theory. Moreover, H_2_ displayed a broad radioprotective ability for the vital lymphocyte of spleen, which was consistent with the other discoveries relating to the hematological system.

As one of the most sensitive targets to radiation induced injuries, sperms are always taken as the endpoint marker for assessing low-dose radiation [23]. Apparently, it is extremely important to protect reproductive system against irradiation induced damage during planned radiation exposure scenarios [31]. Therefore, we conducted the sperm abnormality assessment as another major indicator. As spermatogenesis cycle of mice is approximately 35 days [47], it was the proper time point for us (9 weeks apart from the first time of exposure and 1 week from the last time) to operate the intact process of sperm forming. Depending on the earlier reports, hydrogen-rich saline was demonstrated to protect the spermatogenesis from the acute irradiation of high doses (5~7 Gy) [7, 12]. By contrast, our study aimed at the low doses of radiation in a fractionated long-time method. Our results showed that H_2_ could ameliorate the radiation-elevated sperm abnormalities in each dose level. Of interest, there were numerous similarities between these findings and those of the CAs, both of which were sensitive and appropriate tools to detect low-dose radiation effects. Subsequently, by observing the testicular histopathology sections we found that testicular microstructures were disrupted by gamma rays as low as 0.5 Gy with sharp response to H_2_ administration (Figure 9). Therefore, the potential of H_2_ for improving the male reproductive damage induced by gamma radiation was also confirmed.

As a novel antiradiation agent, molecule H_2_ has drawn great attention of radiobiologists. To our excitement, H_2_ showed an extensive radioprotective ability against various sorts of radiation injuries with lots of advantages such as strong penetrating ability and easy availability rather than any known adverse reactions. So there comes an urgency to develop H_2_ into the mature clinically applied medicine, which requests advanced mechanistic researches as well as considerably more large clinical studies on this amazing molecule.

## 5. Conclusion

In summary, exposing to radiation at this simulated experiment (0.5 Gy, 1 Gy, and 2 Gy fractionated by 16 times) impaired a variety of behavioral and physiological functions, and this research demonstrates that H_2_ could provide an overall radioprotective effect for the damage mentioned above. In addition, this kind of protection is beneficial to many sorts of injured tissues, rather than system-specific or tissue-specific. It is possible that H_2_ exerts the protection by scavenging irradiation enhanced ROS (∙OH and ONOO^−^), but the exact mechanism of the process is urgent to be clarified in the future. Our finding suggested that H_2_ could exert comprehensive beneficial effects on LDLTR radiated mice.

## Figures and Tables

**Table 1:** Chromosome aberrations in bone marrow cells from LDLTR radiated mice.

Dose (Gy)	Treatment	TCC	TAM	TAC	Fragments		Gaps		Dicentrics		RT	DLE	EX	ACR (%)^*∗*^	
					CMT	CTT	CMT	CTT	CMT	CTT				Mean	SD
.0^a^	Nonhydrogen	828	144	148	84	18	12	16	4	6	6	0	2	17.420	3.097
	Hydrogen	842	146	148	84	12	4	14	14	10	10	0	0	17.380	1.731

.1^ab^	Nonhydrogen	848	164	164	98	22	14	12	4	10	2	2	0	19.349	1.758
	Hydrogen	852	152	154	108	16	8	8	8	4	2	0	0	17.865	2.852

.5^ab^	Nonhydrogen	898	192	192	130	26	10	4	18	6	2	2	0	21.390	4.122
	Hydrogen	840	144	146	78	20	8	16	10	8	6	0	0	17.117	1.135

1.0^b^	Nonhydrogen	826	192	198	104	28	14	26	4	12	6	2	2	23.222	1.038
	Hydrogen	830	152	154	70	32	4	12	6	12	18	0	0	18.286	2.016

2.0^c^	Nonhydrogen	834	296	298	170	50	18	16	20	8	16	0	0	35.485	3.298
	Hydrogen	858	244	244	134	22	8	30	10	26	8	4	2	28.350	3.207

All sorts of CAs are scored and displayed here. In each group, eight mice were included for this evaluation, with at least 100 metaphases examined for each of them (*n* = 8). Dose: radiation doses exposed to animals; treatment: whether treating the subjects with hydrogen or not; TCC: total counted cells. TAM: total aberrant metaphases. TAC: total aberrant chromosomes. CMT: chromosomal type; CTT: chromatid type. RT: Robertsonian translocation. DEL: deletion. EX: exchange.

^*∗*^Aberrant cells rate were compared with the method of two-way ANOVA. As for the radiation factor, *F* = 81.457 and *p* = 0.000; hydrogen: *F* = 37.349 and *p* = 0.000. Furthermore, there existed statistically interaction between radiation and hydrogen treatment (*F* = 4.662, *p* = 0.002). The LSD-test was selected for the subsequent comparisons between each radiation dose group.

The aberrant cells rates of groups that have totally different superscripts are significantly different from each other (*p* < 0.05 as significant, SNK test was used here).
